# Diagnosis of disseminated cryptococcosis via iliac bone marrow aspirate analysis

**DOI:** 10.1016/j.idcr.2024.e02136

**Published:** 2024-12-16

**Authors:** Weibu Chen, Xueyan Chen

**Affiliations:** Laboratory Department, Shenzhen People's Hospital, The First Affiliated Hospital, Southern University of Science and Technology, Shenzhen, Guangdong, PR China; Clinical Laboratory, Department of Clinical Laboratory, Shenzhen Longhua District People's Hospital, Shenzhen, PR China

## Abstract

HIV infection frequently affects multiple systems, with hematological manifestations being the most prevalent. In some cases, cryptococcosis serves as the initial manifestation and a cause of infection involving HIV-positive patients. This case report describes a patient with thrombocytopenia who incidentally discovered *Cryptococcus* infiltrating the bone marrow upon bone marrow smear examination, highlighting that examining bone marrow is essential in diagnosing pancytopenia resulting from opportunistic fungal infections like cryptococcosis, especially in individuals with compromised immune systems.

## Introduction

HIV infection frequently affects multiple systems, with hematological manifestations being the most prevalent [1]. The etiologies of hematological abnormalities may be the disease itself, drug effects, secondary malignant neoplasms, or opportunistic infections [2]. In some cases, *cryptococcal* disease (cryptococcosis) serves as the initial manifestation and one of the causes of infection involving HIV-positive patients. The diagnosis of cryptococcosis relied on assessing the titer of cryptococcal antigen present in both serum and cerebrospinal fluid (CSF). Culturing this microorganism is the gold diagnostic method. This case report describes the incidental detection of cryptococcal infection via bone marrow smear in an HIV-positive patient with thrombocytopenia and splenomegaly. We present a case illustrating that examining bone marrow is essential in diagnosing pancytopenia resulting from opportunistic fungal infections like cryptococcosis, especially in individuals with compromised immune systems.

## Case presentation

A 31-year-old Chinese male was referred to our hematology department from his local hospital for the evaluation of thrombocytopenia. The patient was in good health in the past, but there was no obvious trigger for anorexia, abdominal and lumbar pain, and activity limitation 10 days ago. The physical examination showed the patient's enlarged spleen extended across the abdominal midline and reached 6 cm below the umbilicus.

Computed Tomography (C.T.) scans of the chest and abdomen indicated the presence of chronic lung infection, a right posterior mediastinal mass (39 * 21 mm), splenomegaly, and a fatty liver. Laboratory findings were a white blood cell count of 5.85 × 10^9^/L, hemoglobin of 71 g/L, platelet count of 23 × 10^9^/L, ferritin of 1221 ng/ml (reference range 23.9–33.6), albumin of 23.6 g/L (R.R. 40–55), complement C3 of 0.55 g/L (R.R. 0.7–1.40), erythrocyte sedimentation rate (ESR) of 50 mm/h (R.R. < 20 mm/h), and positive direct antiglobulin test. Despite his denial of past infection, the HIV antibody test conducted on the second day of admission yielded a positive result, and Western blot confirmation tests validated the positivity a week later. The CD4+ and CD8+ T lymphocyte counts were 58 cells/μL (1.87 %) and 2303 cells/μL (74.39 %).

**Fig. 1:**
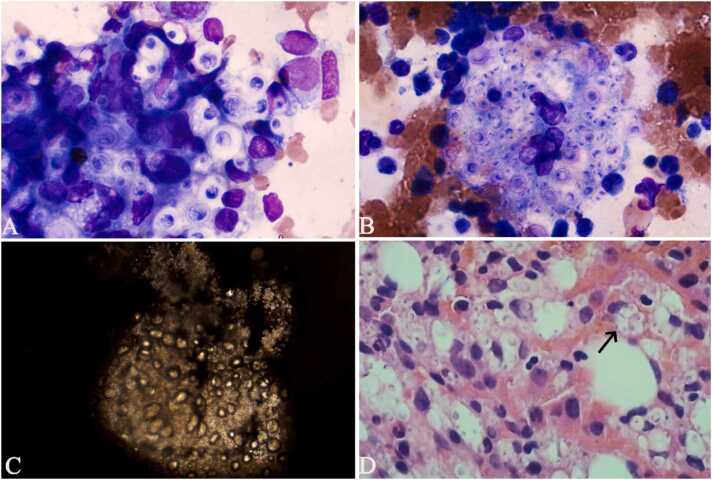
**A–B** The bone marrow aspirate showing numerous phagocytes with many round or oval organisms within the cytoplasm (Wright-Giemsa staining × 100 objective); **C** India ink staining of bone marrow smear revealing transparent round or ovoid thick capsule yeast (India ink staining × 10 objective); **D** Histopathological results of bone marrow revealed yeast (indicated by black arrow, Hematoxylin and eosin staining × 100 objective).

To ascertain the cause of thrombocytopenia and splenomegaly, the patient underwent a bone marrow aspiration. The bone marrow aspirate showed numerous phagocytes with many round or oval organisms within the cytoplasm, accompanied by a transparent and hypertrophic capsule enclosing the microorganisms **(**Fig. 1 **A)**. These organisms are arranged in clusters in phagocytes, easily identifiable in the thicker sections of the smear **(**Fig. 1 **B)**. Additional Indian ink staining was positive, showing transparent round or ovoid thick capsule spores **(**Fig. 1 **C)**. The iliac bone marrow trephine biopsy revealed yeast in the bone tissue, along with infiltration of chronic inflammatory cells in the surrounding tissues **(**Fig. 1 **D)**. Additional serum cryptococcal antigen testing produced positive outcomes. High-throughput sequencing of pathogenic microbial nucleic acids uncovered that the sequence count for *Cryptococcus neoformans* was 135,273, with a relative abundance of 99.73 %. The culture of blood was positive for *Cryptococcus*. The clinicians did not request additional special staining examination for histopathological biopsies because they had already established the basis of cryptococcus infection. Because of the refusal to undergo lumbar puncture, the patient did not undergo the test of cerebrospinal fluid (CSF). The ultimate diagnosis of disseminated cryptococcosis relies on a combination of serum cryptococcal antigen, culture, bone marrow smear analysis, metagenomic next-generation sequencing (mNGS), and pathological biopsy outcomes. Considering that the patient carried a human immunodeficiency virus, we transferred the patient to an infectious disease hospital for further treatment according to legal requirements. However, the patient has remained out of contact since his discharge.

## Discussion

This case's uniqueness lies in identifying *C. neoformans* in bone marrow aspirate, the first clue for diagnosing disseminated cryptococcosis. Cryptococcosis is a significant contributor to morbidity and mortality among immunodeficient individuals, and notably, it is the second most prevalent fungal infection in people with acquired immune deficiency syndromes [3]. Cryptococcosis commonly affects the lungs, central nervous system, or skin [4], while bone marrow involvement is rare. The most common manifestation of AIDS-related cryptococcosis is fungal meningitis. There have only been a few reported cases of bone marrow cryptococcosis, so the exact incidence is uncertain. Patients usually exhibit symptoms associated with these specific sites. Diagnostic clues for bone marrow cryptococcosis emerge from pancytopenia [5], [6], [7], [8], [9]. The relationship between *Cryptococcus* infection and cytopenia remains unclear, as cytopenia often occurs in advanced HIV infection, even in the absence of bone marrow infiltration. *Cryptococcus* synergize with HIV to induce cytopenia [7]. The polysaccharide capsule of *Cryptococcus* has an immunosuppressive effect, decreasing CD4 count [10]. Cryptococcus's activating macrophages in the bone marrow inhibit hematopoiesis, and research indicates that fungal extracts can suppress bone marrow proliferation in leukemia patients [5]. Cryptococcus-related hemophagocytosis, as seen in our case, can potentially lead to cytopenia.

Laboratory diagnosing marrow cryptococcosis involves the detection of cryptococcal antigens, direct microscopic examination, and fungal culture [11]. The serum cryptococcal antigen test is sensitive and detectable before symptom onset. High titers indicate disease progression to the late stage. Many methods have been used in clinical labs to visualize *Cryptococcus* effectively. Indian ink staining is explicitly used for CSF analysis and is unsuitable for other bodily fluids like urine, sputum, or bronchoalveolar lavage fluid. Discerning leukocytes and other interferences from yeast necessitates experience. Histopathological examination, including biopsy and cytology, widely applies to tissues, biological fluids, bronchoalveolar lavage, and fine needle aspiration. Hematoxylin and eosin staining typically showed eosinophilic or slightly basophilic yeast with a halo around its capsule. Certain specialized stains can aid in the identification of *Cryptococcus.*, including Mucicarmine and Alcian dyes for fungal capsule staining, Gomori methenamine silver, periodic acid Schiff, and Calcofluor white for blue-staining fungal cell walls. The fungal culture of Cryptococcus is the gold standard diagnostic method.

mNGS technology has gained increasing utilization in clinical infectious disease diagnosis, especially in cases where cultured or conventional methods fail to identify the causative pathogens. Studies have shown that the sensitivity of mNGS for diagnosing cryptococcal infection is only 44.29 %, significantly lower than the cryptococcal antigen assay, which has a sensitivity of over 95 % [12]. The low positivity rate of *Cryptococcus* is primarily due to its thick cell wall, which often remains incompletely destroyed, preventing the release of nucleic acid [13]. While mNGS lacks advantages in sensitivity and cost, it can promptly report the identification of microbial populations within 24 h. Considering the potential for false negatives in cryptococcal antigen detection and inconclusiveness in biopsies, a positive mNGS result can provide valuable confirmation for clinicians to identify cryptococcal infection and facilitate prompt diagnosis.

Bone marrow is the focal point where the combined effects of infection, drugs, and chronic diseases converge [1]. The literature has reported the presence of characteristic yet nonspecific morphological abnormalities in the bone marrow of AIDS patients, including myelodysplasia, dyserythropoietic, bone marrow fibrosis, plasmacytosis, and granuloma [14]. In this case, the bone marrow exhibited plasmacytosis, constituting 20 % of the nucleated cells. Clinicians often perform bone marrow aspiration to ascertain the etiology of thrombocytopenia, secondary infections, and unexplained fever among AIDS patients.

## Conclusion

Therefore, when conducting a cytological examination of bone marrow aspirate smears, particularly for AIDS patients, it is imperative to carefully inspect for any unusual inclusions within histiocytes to avoid overlooking the diagnosis of an opportunistic infection.

## CRediT authorship contribution statement

**Xueyan Chen:** Methodology, Investigation, Formal analysis. **Weibu Chen:** Conceptualization.

## Author contribution

All authors contributed to the paper's conception and design. Weibu Chen collected clinical and histological data. Xueyan Chen performed histological diagnosis and study design. Xueyan Chen wrote the manuscript draft, and all authors read and approved the final manuscript.

## Author Statement

I have made substantial contributions to the conception or design of the work and interpretation of data for the work.

I have revised it critically for important intellectual content, and I agree to be accountable for all aspects of the work in ensuring that questions related to the accuracy or integrity of any part of the work are appropriately investigated and resolved.

All persons who have made substantial contributions to the work reported in the manuscript, including those who provided editing and writing assistance but are not authors, are named in the Acknowledgments section of the manuscript and have given their written permission to be named. If the manuscript does not include Acknowledgments, it is because the authors have not received substantial contributions from nonauthors.

## Consent

Not applicable.

## Ethical approval

The acquisition of the sample and performance of the study was approved by the ethics review committee of Shenzhen Longhua District People's Hospital, Shenzhen, Guangdong, P.R. China. (Approval no.: 077)

## Funding

No funding is available.

## Declaration of Competing Interest

The authors declare no competing interests.

## Data Availability

The data is available upon request. Please get in touch with the corresponding author, Xueyan Chen.
